# Assessment of prehospital tracheal intubation technique using initial direct laryngoscopy during videolaryngoscopy: randomized controlled simulated trial

**DOI:** 10.1186/s12873-025-01266-0

**Published:** 2025-07-01

**Authors:** Cédric Cibotto, Mathieu Pasquier, Nicolas Beysard, Frédéric Rouyer, Olivier Grosgurin, Laurent Bourgeois, Elio Erriquez, Ely Braun, Birgit Andrea Gartner, Thibaut Desmettre, Laurent Suppan

**Affiliations:** 1 Division of Anaesthesiology, Department of Acute Care Medicine, Geneva University Hospitals, Rue Gabrielle-Perret-Gentil 4, Geneva, 1205 Switzerland; 2 Department of Anaesthesiology, Pharmacology, Intensive Care and Emergency Medicine, Faculty of Medicine, University of Geneva, Geneva, Switzerland; 3 Department of Emergency Medicine, Lausanne University Hospital, University of Lausanne, Rue du Bugnon 46, Lausanne, 1011 Switzerland; 4 Faculty of Biology and Medicine, University of Lausanne, Rue du Bugnon 21, Lausanne, 1011 Switzerland; 5 Division of Emergency Medicine, Department of Acute Care Medicine, Geneva University Hospitals, Rue Gabrielle-Perret-Gentil 4, Geneva, 1205 Switzerland; 6 College of Higher Education in Ambulance Care, ESAMB - École Supérieure de Soins Ambulanciers, Geneva, Switzerland; 7 DMA Département de Médecine Aiguë, Hôpitaux Universitaires de Genève (HUG), Service d’Anesthésiologie, Rue Gabrielle-Perret-Gentil 4, Genève, 1205 Suisse

**Keywords:** Advanced airway management, Direct laryngoscopy, Tracheal intubation, Prehospital, Videolaryngoscopy

## Abstract

**Background:**

In critically ill patients, tracheal intubation may be required in the prehospital setting, where airway management presents unique technical and logistical challenges. While videolaryngoscopy has emerged as a potential alternative to direct laryngoscopy by providing a better and easier visualization of the glottis, the improved view of anatomical structures does not necessarily correlate with successful tracheal tube placement. Intubation may be harder because novice providers performing videolaryngoscopy may only look at the screen and only obtain a two-dimensional representation of the patient’s airways. By directly visualizing the airways, these providers may obtain a better 3D apprehension and an improved mental visualization of the patient’s anatomy. We compared the impact of an unrestricted videolaryngoscopy use with a sequence consisting in direct visualization of the airway followed by videolaryngoscopy (“Direct Laryngoscopy-to-VideoLaryngoscopy sequence” or “DL-VL sequence”) on time to intubation among novice providers.

**Methods:**

This was a parallel group simulated randomized controlled superiority trial. Participants were medical students or junior residents with an experience of less than 10 intubations. After a presentation and workshop on direct laryngoscopy and videolaryngoscopy, participants were randomized in two groups. In the control group, participants were free to use of the videolaryngoscope as they intended. In the other group (DL-VL sequence), participants were told to perform an initial direct laryngoscopy without looking at the video screen until they reached the epiglottis. All intubations were conducted in a simulated prehospital environment, with a high-fidelity manikin placed supine on the floor. Each participant performed three intubations of increasing levels of difficulty. The primary outcome was the time to intubation. Secondary outcomes included first-pass success, time to ventilation, and number of intubation attempts. The chi-squared test was used to compare categorical variables while the t-test was used to compare continuous variables.

**Results:**

Time to intubation was shorter in the control group (22±8 s vs. 27±11 s, *p* < 0.001). This difference was consistent in all levels of difficulties. First-pass success rates were similar (99/111, 89% in the control group vs. 85/105, 81%, *p* = 0.089). Time to ventilation was significantly shorter in the control group (37±9 vs. 41±11 s, *p* = 0.008). The mean number of intubation attempts was similar between groups (*p* = 0.231).

**Conclusion:**

In this simulated study among novice providers, direct airway visualization prior to videolaryngoscopy did not improve time to intubation or to ventilation.

**Trial registration:**

ClinicalTrials.gov, Registration Number: NCT06918717, registered on April 8th, 2025. Retrospectively registered.

**Supplementary Information:**

The online version contains supplementary material available at 10.1186/s12873-025-01266-0.

## Background

Advanced airway management using endotracheal intubation (ETI) can be required in numerous medical and trauma emergencies encountered in prehospital care settings [1–3].

Videolaryngoscopy (VL) is an increasingly used technique in these settings. It provides an indirect view of the glottis without requiring alignment of the upper airways. This feature enhances glottic view, leading to fewer failed attempts and to less oesophageal intubations. It is associated with fewer complications than direct laryngoscopy (DL) [4–6].

Videolaryngoscopes mounted with a reusable standard geometry blade (Macintosh-like) provide the flexibility for dual use, allowing both direct and indirect laryngoscopy. This adaptability enhances the provider’s capacity to tailor their intubation technique based on the clinical scenario or on their personal abilities. There is significant variability among providers in the time spent looking at the screen during intubation with a Macintosh-like videolaryngoscope. Frequent gaze switches between the patient and the display are associated with a lower probability of first-pass success (FPS) [7].

The use of VL is also recommended in the prehospital field [8]. Despite achieving improved laryngeal visualization and a lower incidence of esophageal intubation compared to DL [9–11], VL does not always translate into higher successful intubation rates. Difficulties with tube guidance through the airways have been identified as contributing factors [11], particularly among non-expert providers [12]. VL has also been associated with longer intubation times [12, 13].

Depending on the local organization, novice providers may encounter intubation situations in a prehospital setting, with the patient lying on the floor. However, this positioning, compared to positioning on a stretcher notably modifies the provider’s visual axis during intubation, thereby altering the procedure [14].

DL and VL are distinct intubation techniques requiring different psychomotor skills and hand-eye coordination. To achieve high success rates, providers with high DL experience require deliberate practice with VL to adjust techniques, as they may face challenges when transitioning to a new intubation procedure [15]. However, by virtue of prior DL experience, these providers usually acquire VL skills rapidly [16].

Novice providers present a steeper learning curve with VL than with DL [17]. This difference may be linked to the greater diffculty in obtaining adequate glottic visualization with DL than with VL.

However, despite an optimal glottic view with VL, correct tube placement can still prove difficult, possibly because of the lack of depth appreciation of three-dimensional (3D) structures. Intubating under indirect visualization requires different psychomotor skills compared to direct visualization, and an improved 3-dimensionnal appreciation of the patient’s airway before attempting VL could enable providers to better apprehend the coordination and hand movements required to correctly guide the tracheal tube toward the vocal cords.

No research has yet examined the impact on intubation performance among novice practitioners when having an initial direct vision of the upper airways before switching to the screen during videolaryngoscopy.

This study spawned from the hypothesis that a direct view of the patient’s airways, until the epiglottis is visualized, could decrease the time to intubation (TTI) by allowing a better 3D apprehension of the patient’s anatomy and an improved mental visualization of the laryngeal structures. Despite a two-step process, a first direct visualization before switching to the VL screen could still shorten the procedure by saving significant time by virtue of improved tracheal tube guidance.

The objective was to determine the impact on TTI by achieving an initial direct vision of the upper airways before using visualization of the videolaryngoscope (“Direct Laryngoscopy-to-VideoLaryngoscopy sequence” or “DL-VL sequence”) compared to a free use of the videolaryngoscope (using screen visualization or not) for tracheal intubation in a simulated prehospital setting.

## Methods

### Study design and setting

**Fig. 1:**
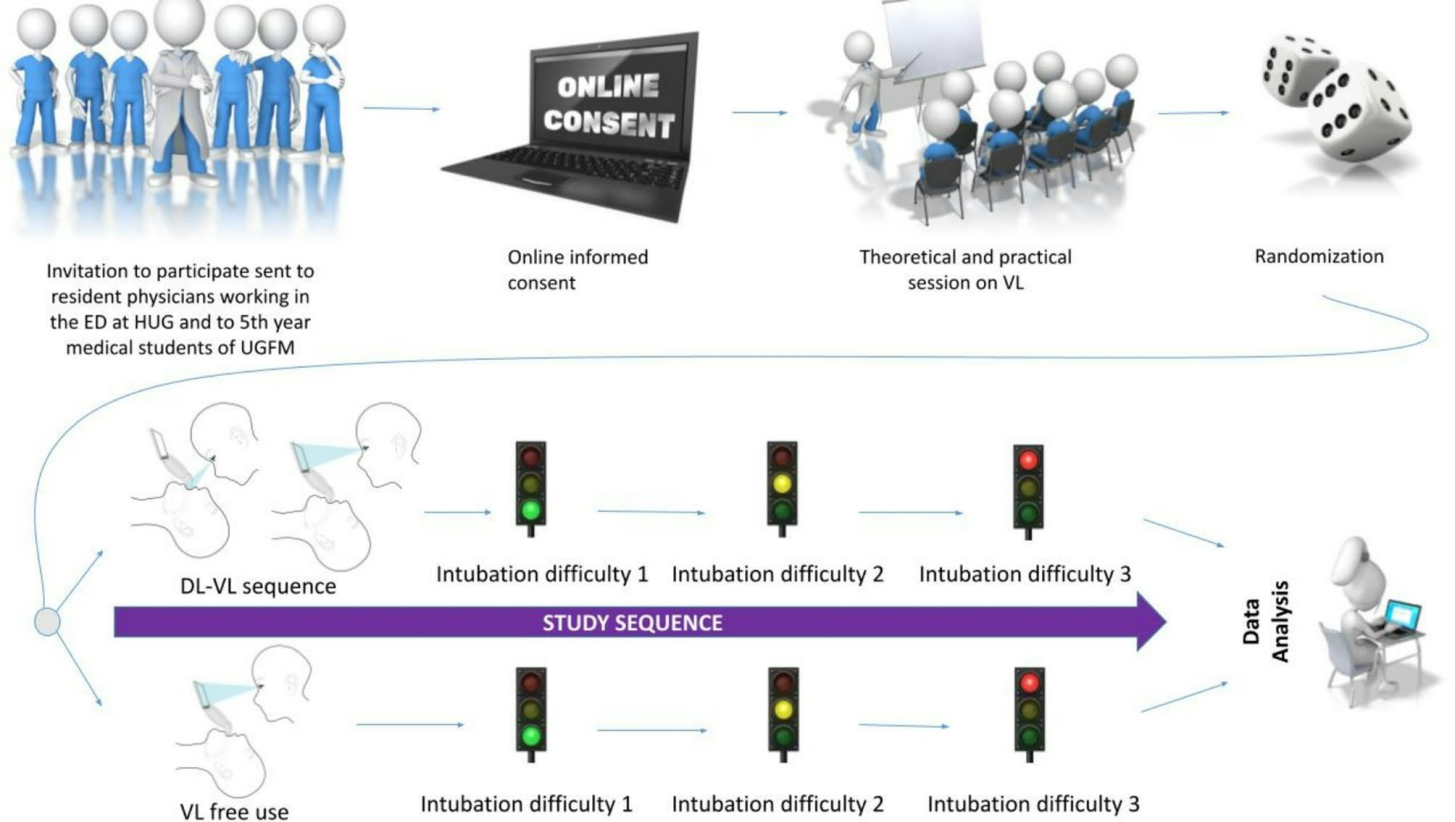
Study design

This was a parallel-group simulated randomized controlled superiority trial whose protocol was designed according to the Standard Protocol Items: Recommendations for Interventional Trials (SPIRIT) statement with evidence-based recommendations [18] [SPIRIT Checklist as Supplementary Table 1]. It is reported according to the updated guidelines of the CONSORT (CONsolidated Standards Of Reporting Trials) statement for reporting parallel group randomized trials [19]. Participant registration, consent and data collection were conducted through an online platform, so relevant elements from the CONSORT-EHEALTH checklist [20] and the Checklist for Reporting Results of Internet E-Surveys (CHERRIES) [21] were also used for reporting. The study design is shown in Fig. 1.

### Participant recruitment and consent

All resident physicians with 1 to 6 years of post-graduate experience working in the Emergency Department (ED) at Geneva University Hospitals (Hôpitaux Universitaires de Genève (HUG)) and penultimate year of medical school students (5th year) studying at University of Geneva Faculty of Medicine (UGFM) were invited to participate. They were considered as novice in airway management if they had performed less than 10 ETIs prior to participating in the study [22].

Before being included in the study, participants were given detailed information, including data use and protection policy. They were asked to provide informed electronic consent. While the general objective was disclosed (studying intubation performance), participants were not provided with the actual outcomes studied and were unaware of the presence of two different groups.

### Study procedures

The research was carried out at the simulation facilities of College of Higher Education in Ambulance Care, Geneva, Switzerland (ESAMB - École Supérieure de Soins Ambulanciers, Genève).

The intubation material consisted of a size 7.0 mm Internal Diameter Tracheal Tube Cuffed (Shiley™, Covidien™) with stylet and a C-MAC Video Laryngoscope (KARL STORZ™ Endoskope, Tuttlingen, Germany) with a reusable metal standard geometry blade (size 4 Macintosh-like).

Participants first followed a theoretical lecture about ETI. They were taught in intubation techniques using both direct and videolaryngoscopy but were not informed on the combining videolaryngoscope use technique. The training was similar for all participants. Then, they attended a practice session using a high-fidelity manikin (Sim-Man 3G Laerdal, Stavanger, Norway) placed on a table, during which an experienced provider demonstrated both intubation techniques while standing. The DL-VL sequence was not demonstrated to the participants during the training session.

After completing this short training program, the study sequence started with the same material and all intubations were conducted in a simulated prehospital environment, with the manikin placed supine on the floor.

In each arm, participants were asked to intubate the manikin three times in progressively increasing levels of difficulty. For the first level of difficulty (easy), no supplemental strain was added to the manikin. For the second level of difficulty (intermediate), a tongue edema was simulated by inflating the manikin’s tongue. For the third level of difficulty (hard), neck movement was limited using the manikin’s editable setting.

Each intubation was video recorded by a GoPro camera (GoPro Hero 10 Black, GoPro Inc™) placed on a tripod. The video laryngoscopies were recorded by the C-MAC.

### Outcomes

The primary outcome was TTI, assessed in seconds from blade insertion at the dental arch to adequate tracheal tube placement through the vocal cords, confirmed by the C-MAC video recording. The duration of the successful intubation attempt was reviewed in the TTI determination, regardless of the number of tries.

Secondary outcomes were the TTI for the first intubation attempt alone, excluding first-attempt failures, the FPS rate, the number of intubations attempts, and the time to ventilation (TTV), defined as the time, in seconds, from blade insertion to successful ventilation confirmed by chest elevation.

An ETI attempt was defined as the insertion of the laryngoscope blade at the dental arch, regardless of whether tracheal tube placement was attempted. A maximum of 60 s was allowed per ETI attempt. In case of esophageal misalignment during the procedure or of withdrawal of the tracheal tube, the intubation attempt was considered failed. The maximum number of ETI attempts was limited to 3 for each difficulty levels. Beyond 3 attempts, the intubation was considered as failed.

Subjective assessments, including perceived difficulty, were appraised using a 5-point Likert scale ranging from “Totally Agree” to “Totally Disagree” (See Supplementary Table 2).

### Sample size

A time difference of 5 s was considered clinically significant. In the prehospital setting, critically ill patients who may not have received adequate preoxygenation are frequently encountered. Some prehospital patients also present with acute respiratory distress. In these circumstances, even a 5-second reduction in time to intubation could potentially prevent or decrease the length and impact of desaturation episodes. Based on existing literature [23], we expected a TTI of 30 s in the “DL-VL” group and 35 s in the control group, with an assumed standard deviation ± 10 s in both groups.

With statistical assumptions of an alpha of 0.05 and for a power of 80%, the estimated number of intubation attempts required to answer our primary outcome was 126 (63 observations per group). Since each participant performed three ETIs, a minimum of 42 participants had to be enrolled.

### Randomization and blinding

Participants were randomized in two groups. A 1:1 block randomization was performed using an online randomizer.

In the control group, participants were not told where to look when performing the intubation. It was a free use of the VL. In the DL-VL group, participants were asked to perform a direct laryngoscopy until they reached the epiglottis before switching to the video screen to perform the tracheal intubation.

Neither the participants nor the on-site investigators could be kept unaware of the sequence allocation. The principal investigator assessed each outcome and was not blinded. However, the precise outcomes were not disclosed to the participants.

### Data collection, extraction and availability

The survey component (Community Surveys Pro 6.3, Shondalai) installed on the online platform was used to ensure data quality through the use of regular expressions (RegEx) and of completeness checks. The same component was used to create an online Case Report Form (CRF). GoPro camera recordings were stored on a secure drive. All data was then imported in Stata 18 (StataCorp, LLC, College Station, TX, USA) for data curation.

### Statistical analysis

Stata 18 was used for statistical analysis. Even though the sample size was deemed high enough for the central limit theorem to apply, the assumption of normality was checked before running statistical tests, and parametric tests were used accordingly. The chi-squared test was used to compare categorical variables while the t-test was used to compare continuous variables.

Intubations were first analyzed separately for each outcome depending on their subgroup of level of increasing intubation difficulty, easy, intermediate and hard. Then, the three subgroups were pooled together for an overall comparison between the two groups.

Binarization was used for the interpretation of questions based on the 5-point Likert scale (“Totally agree” was pooled with “Agree” and “Disagree” was pooled with “Totally Disagree”).

P values < 0.05 were considered statistically significant. Missing data were excluded without any adjustment or imputation.

## Results

A total of 72 participants were enrolled. We exceeded the minimum planned enrollment, aiming for more precise results. The recruitment period took place between September 2023 and April 2024.

The demographic characteristics of participants between groups are detailed in Table 1. Multiple specialties could be selected; therefore, the total percentage may exceed 100%. Emergency medicine is not comprised as an option in the desired medical specialties, as it is not yet recognized as an independent specialty in Switzerland.

**Table 1:** Demographic characteristics of participants

	Control group (*N* = 37)	“DL-VL” group (*N* = 35)
Age - Mean (+/- SD)	25 (+/- 3)	27 (+/- 3)
Sex - N (%)		
Male	11 (30)	17 (49)
Female	26 (70)	18 (51)
Highest educational level - N (%)		
Medical degree	18 (49)	20 (57)
5th year Medical Student	19 (51)	15 (43)
Number of years of post-graduate practice - Mean (+/- SD)	1.2 (+/- 1.5)	1.2 (+/- 1.4)
Desired Specialty - N (%)		
Internal Medicine	18 (48)	24 (68)
Intensive Care Medicine	5 (13)	2 (5)
Anesthesiology	6 (16)	6 (17)
Surgery	5 (13)	1 (2)
Pediatrics	3 (8)	2 (5)
Other	11 (29)	7(20)

Overall, TTI was shorter in the control group (22±8 s) compared to “DL-VL” group (27±11 s), all intubations considered (*p* < 0.001) (Table 2). Detailed results by intubation difficulty level are reported in Table 3.

**Table 2:** Differences between the control and the “DL-VL” groups regarding time to intubation, first pass success, time to intubation for the first intubation attempt and overall time to ventilation

	Overall		
	« DL-VL »	Control	*P*-value
Time to Intubation (seconds, mean±SD)	27±11	22±8	< 0.001
First Pass Success – N (%)	85/105 (81)	99/111 (89)	0.089
Time to Intubation - First ETI attempt (seconds, mean±SD)	26±10	22±8	0.001
Time to Ventilation (seconds, mean±SD)	41±11	37±9	0.008

**Table 3:** Differences between the control and the “DL-VL” groups regarding time to intubation, first pass success, time to intubation for the first intubation attempt and time to ventilation, by intubation difficulty level

	Easy			Inter-mediate			Hard		
	« DL-VL »	Control	*P*-value	« DL-VL »	Control	*P*-value	« DL-VL »	Control	*P*-value
Time to Intubation (seconds, mean±SD)	27±11	23±8	0.042	28±12	22±8	0.017	26±9	20±8	0.008
First Pass Success – N (%)	28/35 (80)	36/37 (97)	0.02	28/35 (80)	34/37 (92)	0.145	29/35 (83)	29/37 (78)	0.631
Time to Intubation- First ETI attempt (seconds, mean±SD)	26±10	23±8	0.139	26±10	22±8	0.162	26±9	20±9	0.007
Time to Ventilation (seconds, mean±SD)	42±11	38±9	0.147	41±12	37±10	0.151	40±10	36±9	0.095

After excluding first-attempt failures, the TTI for the first intubation attempt alone was shorter in the control group. Overall, there was no significant difference regarding the FPS rate. It was however significantly higher in the control group for the first difficulty level compared to the two more difficult subgroups.

The mean number of attempts was similar between the control group and the “DL-VL” group in all the three subgroups of increasing difficulty, respectively (Easy 1.0±0.0 vs. 1.3±0.1, *p* = 0.06, Intermediate 1.1±0.0 vs. 1.3±0.1, *p* = 0.299 and Hard 1.3±0.1 vs. 1.2±0.1, *p* = 0.383), as well as in overall (1.1±0.0 vs. 1.3±0.1, *p* = 0.231).

Overall TTV, was shorter in the control group (37±9) compared to “DL-VL” group (41±11), all intubations considered (*p* = 0.008). There was however no difference regarding TTV between difficulty subgroups (Easy *p* = 0.147, Intermediate *p* = 0.151 and Hard *p* = 0.095).

Subjective assessments did not reveal any significant difference in participant perception of technique difficulty. Binarized results for the question 1 “I found intubation easy” are displayed in Table 4 and binarized results for the question 2 “I felt comfortable intubating this way” are displayed in Table 5. Corresponding graphs can be found in the Supplementary Material (see Supplementary Figs. 1 and 2).

**Table 4:** Question 1 binary results: “I found intubation easy”

	« DL-VL » group	Control group	*P*-value
Agree, easy attempt	27	34	0.447
Disagree, easy attempt	2	1	
Agree, intermediate attempt	18	25	0.446
Disagree, intermediate attempt	7	6	
Agree, hard attempt	15	16	0.417
Disagree, hard attempt	12	8	
Agree, overall	60	75	0.138
Disagree, overall	21	15	

**Table 5:** Question 2 binary results: “I felt comfortable intubating this way”

	« DL-VL » group	Control group	*P*-value
Agree, easy attempt	28	31	0.107
Disagree, easy attempt	0	3	
Agree, intermediate attempt	25	28	0.279
Disagree, intermediate attempt	6	3	
Agree, hard attempt	16	21	0.256
Disagree, hard attempt	8	5	
Agree, overall	69	80	0.369
Disagree, overall	14	11	

## Discussion

In this study, direct visualization of the upper airways by novice providers prior to attempting ETI resulted in longer times to intubation and to ventilation and did not improve FPS. These results do not support our initial hypothesis that direct visualization of the patient’s airways, until the epiglottis is reached, could decrease TTI by allowing a better 3D apprehension of the patient’s anatomy through improved mental visualization of laryngeal structures.

With an average of 27 seconds, the TTI for the “DL-VL” group remained well within the 60-second time limit permitted per attempt. However, there was an increase of 4 to 6 seconds in the “DL-VL” group compared to the control group. This time extension is noteworthy given the short duration of the procedure and could have a significant clinical effect, especially in patients with acute respiratory distress, where optimizing preoxygenation and reducing the apnea period are crucial [24, 25].

These results are similar to those reported in the literature under close conditions. It should be noted that the definition of TTI in these studies was different, as time was measured until chest elevation was established—an equivalent to TTV in our study. For example, Kim et al. reported a TTI of 21.39 s using the GlideScope Ranger among novice paramedics [26], while Chew et al. observed a TTI of 38.5 s using a McGrath with a Macintosh-like blade by novice providers to intubate a manikin with simulated difficult airways [23].

Even though our hypothesis was rejected in the conditions used to carry out this study, it may still be interesting to consider in actual clinical settings. Indeed, the simulated prehospital setting may not reflect an actual clinical situation due to simulation limitations [27–31]. In addition, the prehospital environment presents unique challenging factors, such as limited space, variable exposure, lighting conditions or distractions at the scene [14, 32, 33]. Other factors include airway obstruction from swelling, blood, or vomit, as well as obesity, facial or neck injuries, and restricted mouth opening or neck movement [14, 32, 34–37]. These factors are difficult to reproduce in simulation and may validate the use of direct laryngoscopy instead of indirect laryngoscopy, which can become futile in such conditions.

Different hypotheses can explain these results. A proper glottic view (i.e. Cormack and Lehane score I or II) might have already been obtained under direct laryngoscopy during the first part of the “DL-VL” sequence. In this case, still performing an indirect laryngoscopy for intubation could have led to a waste of time since the provider could already have been able to intubate the trachea without using the video.

Another assumption is that the mere visualization of the epiglottis instead of focusing on the glottis before using the screen may be insufficient to develop a clear mental understanding of the procedure, ultimately limiting its benefits.

Regarding the secondary outcomes, TTV was also shorter in the control group compared to the “DL-VL” group, all intubations considered. We observed a three-second difference between the two groups. Similar to TTI, this time difference in TTV may have clinical importance in cases of acute hypoxemia.

The FPS rate was similar between the two groups, with results close to those found in the literature [35]. The FPS rate remained stable in the “DL-VL” group despite increasing difficulty levels, whereas it decreases in the control group as difficulty rises.

Subjective assessments showed a global satisfaction with the use of the videolaryngoscope, whether in free use or during the double sequence. Notably, participants indicated a favorable opinion of the double sequence technique, valuing it comparatively to the free use of videolaryngoscopy, despite its greater difficulty of use and prolonged time to intubation.

Apart from the aforementioned limitations, others must be acknowledged. Indeed, the participants in our study were exclusively novice providers, and the results obtained through this study cannot apply to all practitioners. The definition of “novice provider” can also be challenged. We found no clear consensus in the literature concerning the definition of an inexperienced provider for intubation; criteria vary across studies, ranging from 0 to 50 intubations, or sometimes even more [38, 39].

Participants executed three repeated intubations, thereby introducing a learning effect that could have skewed the results, although a difference between the two groups was already apparent during the first intubation attempt in the easy difficulty level. Also, using a fixed order of difficulty progression may have induced a confounding effect between learning and difficulty level, as participants may have improved their technique with each successive attempt regardless of the assigned method. Furthermore, the investigators could not blinded because of the study design and were therefore aware of the group allocation both on site and during outcome assessments.

In addition, even though a literature review revealed no clear cut-off defining clinically meaningful TTI differences in this specific context, the actual clinical significance of a 5-second mean difference in the primary outcome could also be considered as a potential limitation. Finally, caution is required when interpreting the secondary outcomes, as the sample size was calculated based on detecting a difference in TTI.

## Conclusions

In this simulated study among novice providers, direct airway visualization prior to videolaryngoscopy lengthened time to intubation and to ventilation and did not improve first-pass success. Because this was an experimental simulation study, these results may not be fully transferable to actual clinical situations. Further research may be warranted to evaluate the applicability of the DL-VL sequence in the more challenging real-world prehospital setting with additional confounding factors and where inter-individual anatomical variations could necessitate optimal 3D appreciation of the airways to improve TTI when using videolaryngoscopy.

## Electronic supplementary material

Below is the link to the electronic supplementary material.

Supplementary Material 1

## Data Availability

The datasets generated and/or analysed during the current study are available in the Yareta© repository, [40]. 10.26037/yareta:sw2t72u4q5f3fcdbm2ou6d5bda.

## Abbreviations

ETI: Endotracheal Intubation
VL: Videolaryngoscopy
DL: Direct Laryngoscopy
FPS: First Pass Success
TTI: Time To Intubation
ED: Emergency Department
HUG: Hôpitaux Universitaires de Genève
UGFM: University of Geneva Faculty of Medicine
TTV: Time To Ventilation
